# IGF-1 overexpression improves mesenchymal stem cell survival and promotes neurological recovery after spinal cord injury

**DOI:** 10.1186/s13287-019-1223-z

**Published:** 2019-05-21

**Authors:** Kyan James Allahdadi, Thaís Alves de Santana, Girlaine Café Santos, Carine Machado Azevedo, Roberta Alves Mota, Carolina Kymie Nonaka, Daniela Nascimento Silva, Clarissa Xavier Resende Valim, Cláudio Pereira Figueira, Washington Luis Conrado dos Santos, Renan Fernandes do Espirito Santo, Afrânio Ferreira Evangelista, Cristiane Flora Villarreal, Ricardo Ribeiro dos Santos, Bruno Solano Freitas de Souza, Milena Botelho Pereira Soares

**Affiliations:** 1 Center for Biotechnology and Cell Therapy, Hospital São Rafael, Salvador, BA Brazil; 2 Gonçalo Moniz Institute, FIOCRUZ, Rua Waldemar Falcão, 121, Candeal, Salvador, Bahia 40296-710 Brazil; 3 Federal University of Bahia, UFBA, Salvador, BA Brazil; 4 National Institute of Science and Technology for Regenerative Medicine, Rio de Janeiro, RJ Brazil; 5 São Rafael Hospital, D’Or Institute for Research and Education (IDOR), Salvador, Brazil

**Keywords:** Spinal cord injury, Bone marrow-derived mesenchymal stem cells, IGF-1, Gene and cell therapy

## Abstract

**Background:**

Survival and therapeutic actions of bone marrow-derived mesenchymal stem cells (BMMSCs) can be limited by the hostile microenvironment present during acute spinal cord injury (SCI). Here, we investigated whether BMMSCs overexpressing insulin-like growth factor 1 (IGF-1), a cytokine involved in neural development and injury repair, improved the therapeutic effects of BMMSCs in SCI.

**Methods:**

Using a SCI contusion model in C57Bl/6 mice, we transplanted IGF-1 overexpressing or wild-type BMMSCs into the lesion site following SCI and evaluated cell survival, proliferation, immunomodulation, oxidative stress, myelination, and functional outcomes.

**Results:**

BMMSC-IGF1 transplantation was associated with increased cell survival and recruitment of endogenous neural progenitor cells compared to BMMSC- or saline-treated controls. Modulation of gene expression of pro- and anti-inflammatory mediators was observed after BMMSC-IGF1 and compared to saline- and BMMSC-treated mice. Treatment with BMMSC-IGF1 restored spinal cord redox homeostasis by upregulating antioxidant defense genes. BMMSC-IGF1 protected against SCI-induced myelin loss, showing more compact myelin 28 days after SCI. Functional analyses demonstrated significant gains in BMS score and gait analysis in BMMSC-IGF1, compared to BMMSC or saline treatment.

**Conclusions:**

Overexpression of IGF-1 in BMMSC resulted in increased cell survival, immunomodulation, myelination, and functional improvements, suggesting that IGF-1 facilitates the regenerative actions of BMMSC in acute SCI.

**Electronic supplementary material:**

The online version of this article (10.1186/s13287-019-1223-z) contains supplementary material, which is available to authorized users.

## Background

Spinal cord injury (SCI) affects millions of people across the globe, frequently leading to paralysis in a productive age, with a significant impact to the affected individual and family, as well as to the healthcare system [1]. Following the primary SCI event, the inflammatory response and oxidative stress can lead to augmented damage—secondary injury—to the tissue proximal to the injured site [2]. The loss of functional neurons and demyelination leads to impaired neural circuitry of the central nervous system (CNS), with limited spontaneous regenerative capacity [3]. Current treatment options are limited to supportive care and injury management, reinforcing the need for the development of novel treatment options that could limit secondary damage and stimulate the regeneration of the damaged spinal cord.

Stem cell therapy is a promising field that has been investigated as a therapeutic option for SCI [4]. While different types of adult, fetal-derived, or embryonic stem cells have been investigated, bone marrow-derived mesenchymal stem cells (BMMSCs) are an attractive option for such therapies, since the cells can be easily obtained and applied in autologous transplantation procedures. BMMSCs can promote SCI recovery through the immunomodulation [5], activation, and homing of endogenous stem/progenitor cells [6] and by the production of growth factors, neuroprotective cytokines, and chemokines [7, 8]. However, the effects of BMMSCs can be limited by poor survival or limited lifespan, following transplantation [5]. Genetic engineering of BMMSCs is a promising approach to improve cell survival and influence surround tissue via paracrine actions, through overexpression of specific growth factors of interest [9].

Insulin-like growth factor 1 (IGF-1) is a protein hormone that is produced and secreted by a variety of cells, including BMMSCs [10]. It is also an important growth factor that participates in the development of the central nervous system, as well as in recovery, following injury or pathological processes [11, 12]. IGF-1 was previously reported to promote oligodendrocyte differentiation and survival during normal development [13], and IGF-1 overexpression leads to increased brain size and myelin content [14]. Moreover, IGF-1 acts as a potent antioxidant [15] and pro-survival [16] factor in the central nervous system. Therefore, the beneficial properties of IGF-1 could be an effective method in the treatment of SCI. We have recently generated and characterized a BMMSC line, genetically engineered to overexpress IGF-1 (BMMSC-IGF1) [9]. In the present study, we evaluated the therapeutic potential of BMMSC-IGF1 in a moderate contusion model of SCI in mice, by histopathological, immunological, and functional evaluation after treatment.

## Materials and methods

### Mesenchymal stem cell (BMMSC) culture

Wild-type BMMSCs were obtained from male GFP transgenic C57Bl/6 mice. A genetically modified BMMSC line with stable overexpression of hIGF-1 (BMMSC-IGF1) was previously generated by the transduction with a lentiviral vector and characterized by our group [9]. BMMSCs were maintained in Dulbecco’s modified Eagle’s medium (DMEM), 10% fetal bovine serum, and 1% penicillin/streptomycin (all from Thermo Fisher Scientific, Waltham, MA, USA) in a humidified incubator at 37 °C and atmosphere with 5% CO_2_, under medium replacement every 3 days for expansion.

### Animals and surgical procedures for laminectomy and spinal cord injury

C57Bl/6 female mice, 8–12 weeks old, were used throughout this study. Animals were raised and maintained at the animal facility of the Center for Biotechnology and Cell Therapy, Hospital São Rafael (Salvador, Brazil), with access to food and water ad libitum. The use of animals and experimental protocols were approved by the local Ethics Committee, which follows NIH guidelines (Hospital São Rafael, 02/12).

For spinal cord injury (SCI) induction, mice were anesthetized using ketamine (80–100 mg/kg IP; Cristalia, Itapira, Brazil) and xylazine (10–12.5 mg/kg IP, Rhobifarma, Hortolândia, Brazil), after which they were subjected to a laminectomy surgery, followed by contusive SCI. This involved identification of the tenth thoracic (T10) vertebra based on anatomical landmarks, followed by a dorsal laminectomy of T10. Following the removal of the T10 lamina, a moderate contusion injury (~ 70 kdyn) was applied to the spinal cord using the Infinite Horizon Impactor (Precision Systems and Instrumentation, Fairfax Station, VA, USA). After SCI, 3 μl of saline, BMMSCs (1 × 10^6^ cells), or BMMSC-IGF1 (1 × 10^6^ cells) were injected (Hamilton Syringe #705) directly into the SCI epicenter, positioned with the use of a stereotaxic instrument (Kopf Instruments, Tujunga, CA, USA), at a flow rate of 1 μl/min (Harvard Apparatus Pump11 Elite). The muscle and skin were closed using 5–0 ethilon sutures (Ethicon-Johnson & Johnson, Somerville, NJ, USA). Bladders were checked twice daily and manually voided when necessary, for the duration of the experiment.

### Functional recovery assessment

Hind limb locomotor recovery was assessed on days 1, 7, 14, 21, and 28 post-SCI, using the Basso Mouse Scale (BMS), an evaluation specifically designed for mouse models of contusive SCI [17]. Each scoring day, two blinded investigators, that were trained and experienced in BMS evaluations, performed the assessment on randomly selected mice in an open field during a 5-min period. A 70-kdyne contusive SCI normally results in immediate paraplegia in mice, demonstrating a nearly complete or complete loss of hind limb movement at 1 day after injury.

Additional functional analysis was performed using DigiGait, an undermount video recording and gait analysis system (DigiGait, Mouse Specifics, Inc. Framingham, MA, USA). Mice with SCI were conditioned to the DigiGait system, daily for 1 week, before actual experiments were conducted.

### Immunohistochemistry and histology

Mice were terminally anesthetized and perfused with saline, then fixed with 4% paraformaldehyde (PFA) in PBS. The spinal cord was removed from the body, maintained overnight in 4% PFA (4 °C) then cryoprotected overnight in 30% sucrose in PBS. The next day, the spinal cord was trimmed down to the injured portion (5 mm of total length) and either (1) embedded in Tissue-Tek, frozen and maintained at − 80 °C, then sectioned at 10 μm or (2) fixed in 10% formalin, paraffin embedded and sectioned at 4 μm.

In frozen spinal cord sections, the following primary antibodies or dye were used: GFP, Ki-67, and cleaved caspase-3. Frozen sections were incubated in primary antibody solution containing chicken anti-GFP (1:800, Life Technologies, Carlsbad, CA, USA) and rabbit anti-mouse Ki-67 (1:800, Thermo Fisher Scientific, Waltham, MA, EUA) or rabbit anti-mouse cleaved caspase-3 (1:400, Cell Signaling, Danvers, MA, USA), diluted in PBS/BSA 1%. After overnight 4 °C incubation, the sections were washed two times for 5 min in PBS Tween 0.05% and repeated in the same manner with PBS. The fluoromyelin staining was performed according to the manufacturer’s protocol (FluoroMyelin green, Invitrogen, Carlsbad, CA, USA). Cryosections were fixed in paraformaldehyde 4% (Electron Microscopy Sciences, Hatfield, PA, USA) for 15 min, and sections were then permeabilized in Triton X-100 0.1% for 10 min. Non-specific protein binding was blocked by incubating the sections in PBS/BSA 5% for 1 h. Sections were then incubated with secondary antibody donkey anti-rabbit IgG Alexa Fluor 568 conjugated (1:1000, Molecular Probes, Carlsbad, CA, USA) and donkey anti-chicken IgG Alexa Fluor 488 conjugated (1:600, Molecular Probes, Carlsbad, CA, USA) for 1 h at room temperature. Nuclei were counterstained with 4,6-diamidino-2-phenylindole (DAPI) (Vector Labs, Burlingame, CA, USA).

Paraffin-embedded sections were performed following standard histological methods, briefly detailed below. Following deparaffinization, rehydration, and heat-induced antigen retrieval, the Mouse-on-mom kit (M.O.M kit, Vector Labs, Burlingame, CA, USA) was used to stain with the following primary antibodies: goat anti-mouse Iba-1 (1:1000, Abcam, Cambridge, UK), mouse anti-PCNA (1:200, Agilent, Santa Clara, CA, USA), rabbit anti-mouse APC (1:200, Santa Cruz, Dallas, TX, USA), rabbit anti-mouse GFAP (1:1000, Agilent, Santa Clara, CA, USA), or rabbit anti-mouse doublecortin (1:200, Abcam, Cambridge, UK). Sections were then incubated with secondary antibody donkey anti-rabbit IgG Alexa Fluor 568 conjugated (1:1000, Molecular Probes, Carlsbad, CA, USA) and streptavidin Alexa Fluor 488 conjugated (1:800, Molecular Probes, Carlsbad, CA, USA) for 1 h at room temperature. Nuclei were counterstained with 4,6-diamidino-2-phenylindole (DAPI) (Vector Labs, Burlingame, CA, USA).

The presence of fluorescent cells was determined by observation using a confocal laser scanning microscope A1R (Nikon, Tokyo, Japan) and processed using Image-Pro Plus version 7.01 (MediaCybernetics, Rockville, MD, USA). Lesion volume measurement was based on GFAP staining, which concentrated on the border of the injured region, combined with fluoromyelin staining of the remaining tissue in the section. Therefore, lesion volume was determined based on the ratio of the injured region (void of stained myelin and marked by GFAP) and uninjured region (area marked by fluoromyelin).

### Estimation of nitrite and lipid peroxidation

At the end of the experimental period, spinal cords were collected. Injured spinal cord segments (T9-T10) were rinsed with ice-cold saline (0.9% sodium chloride) and homogenized in chilled phosphate buffer (pH 7.4). Thus, the homogenate obtained was used to assay lipid peroxidation and nitrite estimations. The malondialdehyde (MDA) content, a marker of lipid peroxidation, was assayed in the form of thiobarbituric acid-reactive substances, as previously described [18]. Briefly, 0.5 ml of homogenate and 0.5 ml of Tris–HCl were incubated at 37 °C for 2 h. After incubation, 1 ml of 10% trichloroacetic acid was added and centrifuged at 1000*g* for 10 min. For every 1 ml of supernatant, 1 ml of 0.67% thiobarbituric acid was added and the tubes were kept in boiling water for 10 min. After cooling, 1 ml of double distilled water was added and absorbance was measured at 532 nm. Thiobarbituric acid-reactive substances were quantified using an extinction coefficient of 1.56 × 10^5^ M^−1^ cm^−1^ and were expressed as nmol of malondialdehyde per mg protein. Nitrite was estimated in the spinal cord homogenate using the Griess reagent and served as an indicator of nitric oxide production. A quantity of 500 μL of Griess reagent (1:1 solution of 1% sulphanilamide in 5% phosphoric acid and 0.1% naphthylamine diamine dihydrochloric acid in water) was added to 100 μL of homogenate and absorbance was measured at 546 nm. Nitrite concentration (μg/ml) was calculated using a standard curve for sodium nitrite.

### RT-qPCR

Total RNA has been extracted using TRIZOL® (Thermo Fisher Scientific, Waltham, MA, USA) following the manufacturer’s instruction. RNA integrity was assayed by 1% agarose electrophoresis, and purity was measured photometrically using the NanoDrop™ 1000 (Thermo Fisher Scientific, Waltham, MA, USA). RNA samples (1 μg per sample) were converted to cDNA using High-Capacity cDNA Reverse Transcription Kit (Thermo Fisher Scientific, Waltham, MA, USA). In order to quantify mRNA expression, *Mrc1* (Mm00485148_m1), *Sod1* (Mm01344233_g1), *Cat* (Mm00437992_m1), *Gpx3* (Mm00492427_m1), and *Nfe2l2* (Mm00477784_m1) Taqman Master Mix and Taqman™ probes were used in a final volume of 10 μL, following the manufacturer’s instruction (all from Thermo Fisher Scientific, Waltham, MA, USA). All RT-qPCR data was normalized with *Gapdh* and *Hprt*. PCR amplification was performed in an ABI7500 Real-Time PCR System (Applied Biosystems, Foster City, CA, USA) under standard thermal cycling conditions. The threshold cycle method of comparative PCR was used to analyze the results [19]. Data was analyzed using GraphPad software version 6.

### Transmission electron microscopy

Twenty-eight days post-lesion, 12 animals (BMMSC-IGF1, *n* = 3; BMMSCs, *n* = 3; saline, *n* = 3; uninjured control, *n* = 3) were terminally anesthetized and perfused with 4% paraformaldehyde and 0.25% glutaraldehyde (Sigma-Aldrich, St. Louis, MO, USA) in 0.1 M sodium cacodylate buffer. Spinal cords were collected and fixed at 4 °C for 24 h in a solution of 2% paraformaldehyde and 2.5% glutaraldehyde in 0.1 M sodium cacodylate buffer. Following fixation, 1 mm injured spinal cord segments (lesion site) were removed and maintained in the fresh fixing solution (above) for 72 h. Segments were then washed with 0.1 M sodium cacodylate buffer and post-fixed in osmium tetroxide (Electron Microscopy Sciences Inc., Hatfield, PA, USA) 1% for 1 h. Segments were dehydration by using a graded series of acetone solutions (from 30 to 100%) before embedding the samples in epoxy resin Polybed812 (Electron Microscopy Sciences Inc., Hatfield, PA, USA). Ultrathin sections were obtained using EM UC7 ultramicrotome (Leica Microsystems, Wetzlar, Germany) and contrasted with uranyl acetate and lead citrate. The sections were analyzed by transmission electron microscope JEM1230 (JEOL, Tokyo, Japan) at 80 kV.

For the analysis of histopathological alterations, 10 images were randomly taken from each animal and analyzed by three experienced investigators in order to visualize the presence of collagen fibers, degeneration vacuoles, and myelinated and demyelinated axons, for quantitative analysis. Magnification for all images was set at × 5000 using Fiji software version 1.51. Additionally, three images from each animal were selected to measure axon diameter (inner diameter of the axon), fiber diameter (inner diameter of the axon plus myelin sheath), myelin thickness, and g-ratio (axon diameter/fiber diameter). For this analysis, images with a greater number of myelinated axons from each animal were selected.

### Statistical analyses

Behavioral data were analyzed using the two-way ANOVA (group and time) followed by Bonferroni’s multiple comparisons. Remaining data were analyzed using Student’s *t* test or one-way ANOVA followed by Tukey or Newman-Keuls multiple comparison tests. All data were analyzed using the GraphPad Prism v.5.0 software (GraphPad Inc., San Diego, CA, USA). Differences were considered statistically significant for *P* values < 0.05.

## Results

### IGF-1 expression increases survival of transplanted BMMSCs and activation of endogenous progenitors in acute SCI

**Fig. 1:**
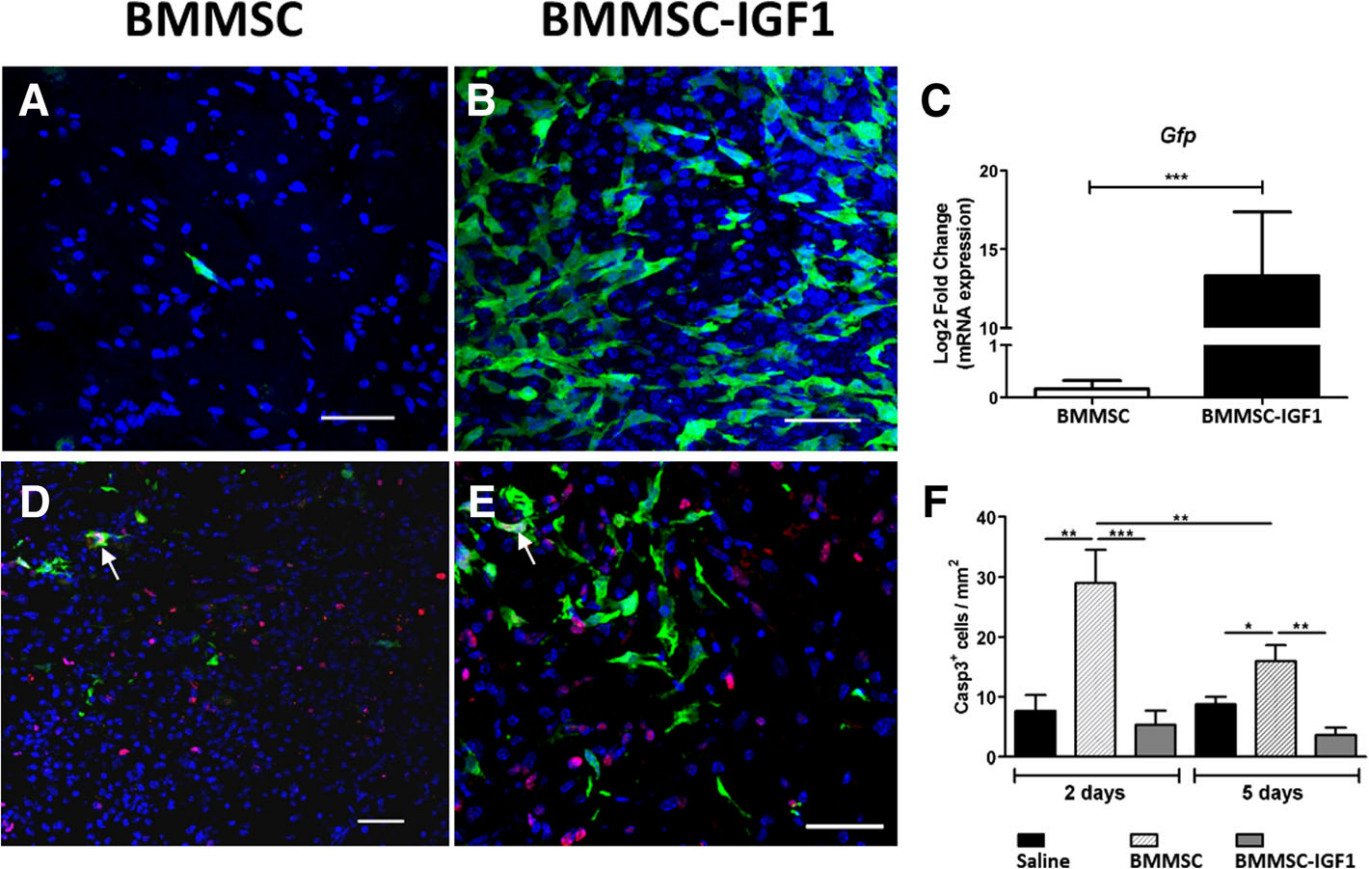
Increased survival of BMMSC-IGF1 and induction of cell proliferation in the injured spinal cord. Presence of GFP^+^ BMMSCs was detected in spinal cord sections of mice transplanted with wild-type BMMSCs (**a**) or BMMSC-IGF1 (**b**), visualized in green by confocal microscopy, 5 days after spinal cord injury and cell transplantation. Nuclei were stained with DAPI (blue). Scale bars = 50 μm. **c** Quantification of GFP mRNA in the injured spinal cord segments, isolated at 5 days post-injury and transplant, measured by qRT-PCR. Confocal microscopy of injured mouse spinal cords, 5 days post-injury and transplanted with wild-type BMMSC (**d**) or BMMSC-IGF1 (**e**), immunostained for the proliferation marker Ki-67 (red) and nuclei stained with DAPI (blue). Scale bars = 200 μm. **f** Quantification of Casp3^+^-labeled cells in spinal cord sections. Values represent mean ± SEM. *P < 0.05; **P < 0.01; ***P < 0.001

After induction of SCI, mice were injected intralesionally with BMMSCs, BMMSC-IGF1, or saline, as shown in Additional file 1: Figure S1. First, we evaluated the presence of transplanted BMMSCs and BMMSC-IGF1 in the injured spinal cord, by tracking GFP^+^ cells. The number of GFP^+^ cells detected in the SCI epicenter 5 days post-injury was higher in mice transplanted with BMMSC-IGF1 than with wild-type BMMSCs (Fig. 1a, b). This was confirmed by GFP gene expression analysis by RT-qPCR, which demonstrated increased GFP gene expression in the spinal cord of mice transplanted with BMMSC-IGF1, when compared to those receiving wild-type BMMSCs (Fig. 1c).

The higher numbers of GFP^+^ cells in the spinal cord of BMMSC-IGF1 mice could be the result of increased cell proliferation or pro-survival actions induced by IGF-1. Therefore, we evaluated if IGF-1 overexpression induces the proliferation of donor and/or recipient cells in the spinal cord, by analyzing the proliferation marker Ki67. Proliferating cells were mostly recipient cells, since GFP^+^ Ki67^+^ cells were rarely observed (Fig. 1d, e) and represented ~ 2.2% of the number of proliferating cells in the spinal cord in the BMMSC-IGF1-treated group. The number of GFP^+^Ki67^+^ cells was similar to BMMSC- and BMMSC-IGF1-treated mice (data not shown). In order to evaluate if IGF-1 expression could increase cell survival by inhibiting apoptosis, we performed immunostaining for cleaved caspase-3 and quantified the number of cells undergoing apoptosis, 2 and 5 days post-SCI (Fig. 1f). We detected a higher number of Casp3^+^ cells in the group treated with wild-type BMMSCs in both time points, with an increased number of Casp3^+^ cells 2 and 5 days after SCI-cell transplantation, in the BMMSC-treated group (Fig. 1f).

**Fig. 2:**
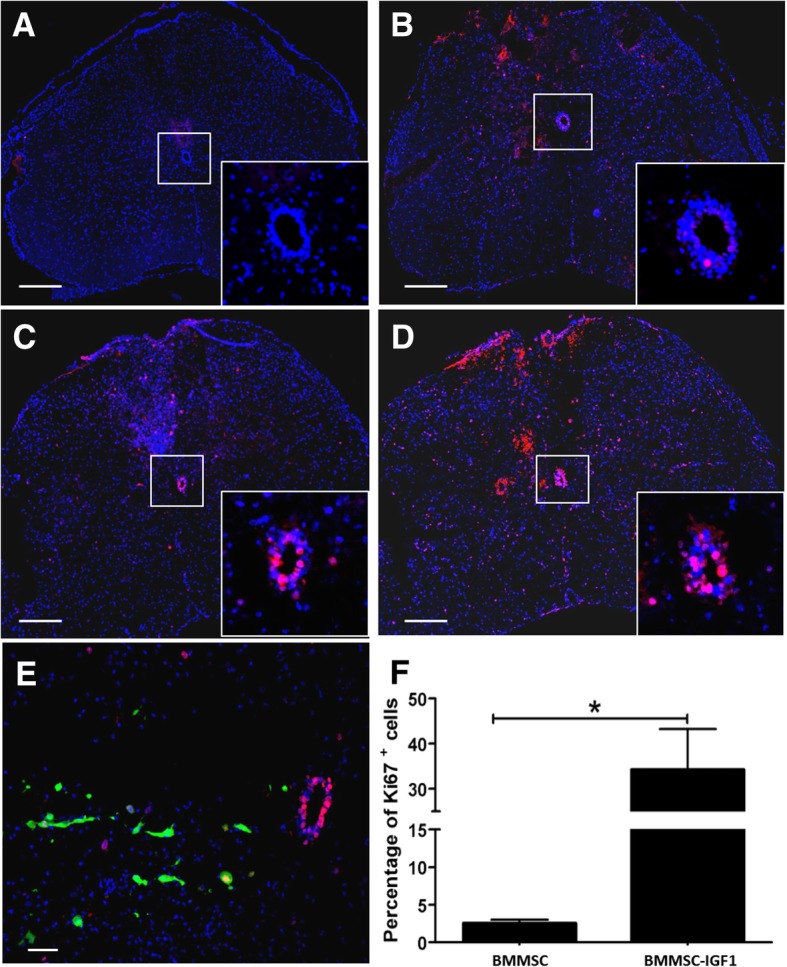
Cellular proliferation in injured mouse spinal cords following BMMSC-IGF transplantation. Spinal cord segments from **a** uninjured mouse and SCI mice treated with **b** saline, **c** BMMSCs and **d**, **e** BMMSC-IGF1, observed by confocal microscopy, 2 days following spinal cord injury and cell transplantation, immunostained for Ki-67. Scale bars = 200 μm (**a**–**d**); scale bar = 50 μm (**e**). Central canal (highlighted/insert, **a**–**d**). **f** Quantitative percentage of Ki-67^+^ in the spinal cord 5 days post-injury. Values represent mean ± SEM. *P < 0.05; **P < 0.01; ***P < 0.001

Proliferating cells (Ki67^+^) were mainly located in the central canal, proximal to the injury area, 2 days after SCI (Fig. 2). Increased proliferation of central canal ependymal cells was observed in mice treated with either BMMSCs or BMMSC-IGF1, when compared to saline-treated and uninjured mice (Fig. 2a–d). Transplanted cells were found proximally to cells proliferating in the central canal (Fig. 2e). At 5 days post-injury, proliferation of central canal cells was reduced and we observed an increased number of proliferating cells surrounding the injury area, which was greater in BMMSC-IGF1-treated mice (Fig. 2f).

**Fig. 3:**
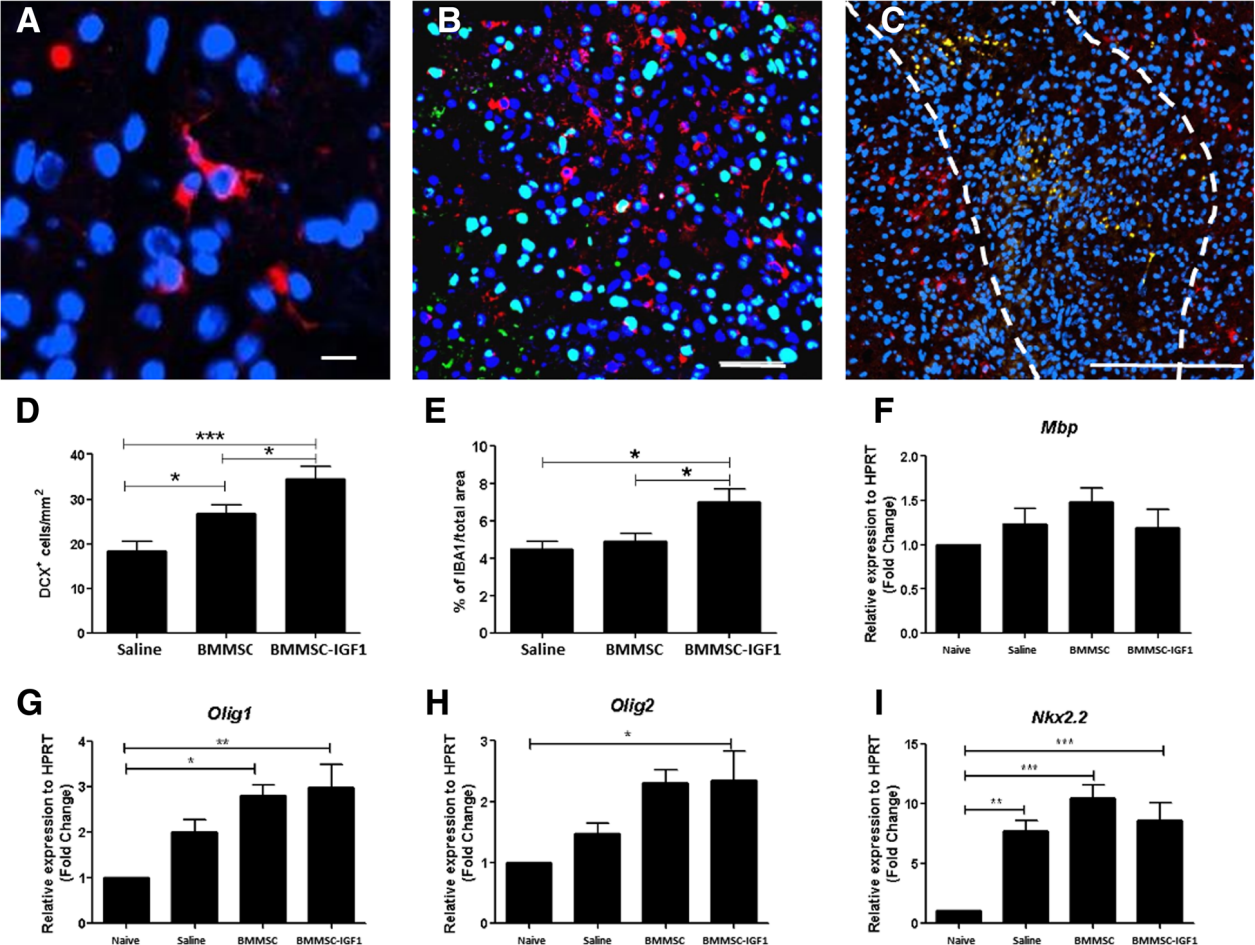
SCI mice treated with BMMSC-IGF1 demonstrate elevated progenitor cell presence, 5 days following SCI and transplant. Injured spinal cord section from BMMSC-IGF1-treated mouse immunostained for **a** DCX (red; scale bar = 10 μm), **b** double immunostained for DCX and PCNA (green; scale bar = 10 μm), and **c** detailed region with DCX (red; scale bar = 100 μm). Quantitative analysis of **d** DCX-positive cells and **e** percentage of IBA1 cells from injured spinal cords. RT-qPCR analysis of expression of **f** Mbp, **g** Olig1, **h** Olig2, and **i** NKx2.2, genes associated with oligodendrocyte progenitor cells differentiation. Values represent mean ± SEM. *P < 0.05; **P < 0.01; ***P < 0.001

Next, we investigated whether BMMSC-IGF1 transplantation was associated with activation of endogenous progenitors. We stained for immature neurons/progenitors (DCX) and observed a greater number of DCX^+^ cells in spinal cords from injured mice transplanted with BMMSC-IGF1 when compared to uninjured, saline-treated, and BMMSCs-treated, 5 days post-injury (Fig. 3a–d). DCX^+^ cells were not co-stained with a proliferation marker (Fig. 3b) and were mainly located in the area surrounding the injury (Fig. 3c). Additionally, BMMSC-IGF1 treatment resulted in increased staining of macrophage/microglia marker (IBA1, Fig. 3e). We also evaluated the expression of markers for oligodendrocyte differentiation—MBP, Olig1, Olig2, and Nkx2.2—by RT-qPCR of the spinal cord tissue (Fig. 3f–i). While MBP gene expression was similar between the groups, Nkx2.2 was increased in all injured spinal cords, and Olig1 and Olig2 levels of expression were increased in BMMSC and BMMSC-IGF1 groups.

### Modulation of inflammatory cytokines and oxidative stress by BMMSC-IGF1

**Fig. 4:**
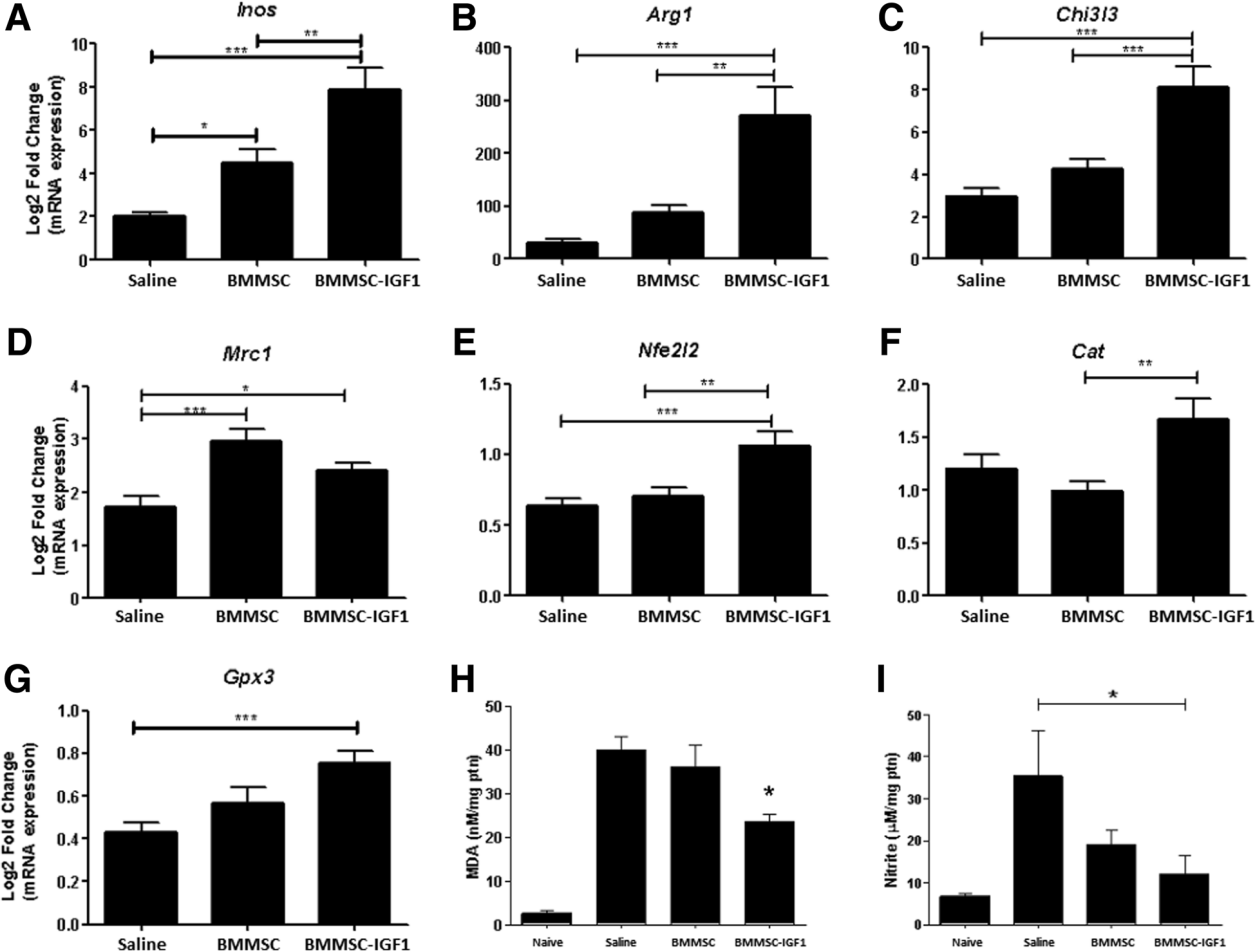
Modulation of inflammatory mediators after SCI and treatment. Transcripts for **a** iNOS, **b** Arg1, **c** Chi3I3, **d** Mrc1, **e** Nfe2I2, **f** Cat, and **g** Gpx3 were determined in SCI segment homogenates from saline (*n* = 5), BMMSC (*n* = 5), or BMMSC-IGF1-treated mice, by RT-qPCR. Values represent means ± SEM. Concentrations of **h** malondialdehyde (MDA), measured by MDA Oxidative Stress Assay, and **i** nitrite, determined by the Griess method, in SCI segment homogenates from naive, (*n* = 5), saline (*n* = 5), BMMSC (*n* = 5), or BMMSC-IGF1-treated mice. Values represent mean ± SEM. *P < 0.05; **P < 0.01; ***P < 0.001

Following the observation of increased Iba1 staining after therapy with BMMSC-IGF1, we evaluated the expression of macrophage/microglia polarization markers, NOS2 (type I activation marker, Inos) (Fig. 4a), as well as type II activation markers Arg1 (Fig. 4b), and Chi3L3 (Fig. 4c), which were found to be increased in BMMSC-IGF1-treated mice, when compared to vehicle or wild-type BMMSCs-treated mice. Mrc1 was increased following treatment with BMMSC and BMMSC-IGF1, when compared to saline (Fig. 4d). Gene expression analysis of factors related to anti-oxidant response showed increased expression of Nfe2L2 (Fig. 4e), Cat (Fig. 4f), and Gpx3 (Fig. 4g) in mice treated with BMMSC-IGF1, when compared to wild-type BMMSC or vehicle treatment. Based on this, we investigated the production of oxidative stress metabolites in the injured spinal cord. MDA content, an indicator of lipid peroxidation, was significantly reduced in the BMMSC-IGF1 group when compared to BMMSC and saline samples (Fig. 4h). Similarly, nitrite concentration was significantly reduced in the BMMSC-IGF1-treated group when compared to saline, but not when compared to BMMSC-treated mice (Fig. 4i).

### Functional improvements in SCI mice treated with BMMSC-IGF1

**Fig. 5:**
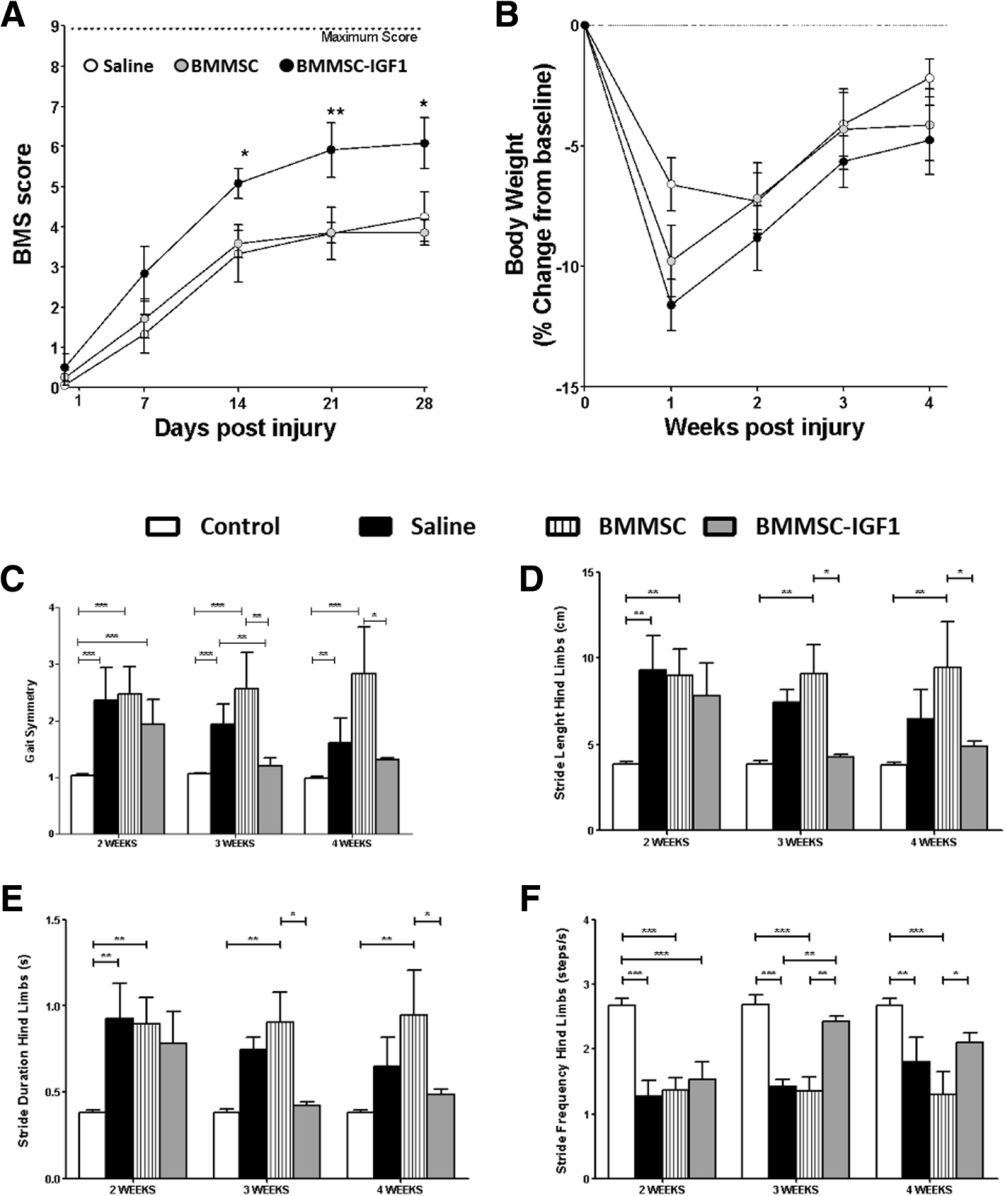
Functional analysis following SCI and treatment. Functional analysis (**a**) of saline-, BMMSC-, and BMMSC-IGF1-treated mice, evaluated weekly from day 1 to 28 days using the Basso Mouse Score (BMS). Body weight (**b**) was assessed with BMS and is represented as percent change in body weight, based on weight prior to injury (baseline). DigiGait-derived functional measurements of **c** gait symmetry, **d** stride length, **e** stride duration, and **f** stride frequency were evaluated at weeks 2, 3, and 4. Values represent mean ± SEM. *P < 0.05; **P < 0.01; and ***P < 0.001

After the conclusion of short-term analyses, we performed experiments to evaluate functional recovery during the 4 weeks following SCI to investigate whether BMMSC-IGF1 therapy resulted in locomotion improvement in SCI mice. First, we applied the Basso Mouse Scale (BMS) score to evaluate the progressive gains in gait, weight bearing, and coordination [17]. BMS scoring analysis was initiated 1 week after SCI and therapy and was repeated weekly over the course of 28 days. Significant gains in BMS were observed in SCI mice treated with BMMSC-IGF1 beginning at week 2 and continued until the conclusion at week 4, compared to BMMSC-treated and vehicle (saline)-treated SCI mice (Fig. 5a). No significant difference was observed between BMMSC- and saline-treated SCI mice. Body weight was simultaneously measured, and no difference between groups was observed post-injury and during the recovery period (Fig. 5b).

Gait analysis was performed using the DigiGait system, which was evaluated 2, 3, and 4 weeks after SCI and treatment. Gait symmetry is evaluated to determine the ratio of forelimb-to-hindlimb stepping, which is approximately 1.0 in uninjured mice. Gait symmetry in BMMSC-IGF1 SCI mice was significantly higher than uninjured mice at week 2; however, no difference was measured during weeks 3 and 4, while saline-treated SCI mice were significantly different from uninjured mice at all time points (Fig. 5c). Various aspects of hindlimb stride were analyzed, including stride length (Fig. 5d), stride duration (Fig. 5e), and stride frequency (Fig. 5f). Significant differences between uninjured and SCI BMMSC-IGF1 mice were measured at 2 weeks, in all parameters (Fig. 5c–f); however, starting at week 3 and continuing to week 4, BMMSC-IGF1 levels were similar to uninjured levels. Saline-treated SCI mice demonstrated improvements in stride length and duration by week 4 (Fig. 5d, e) but had no significant gains in stride frequency (Fig. 5f). Representative images of an uninjured mouse and SCI mice after 4 weeks of treatment with saline, BMMSC, and IGF1, demonstrating hindlimb positioning and gains measured in DigiGait analysis (Additional file 2: Figure S2 and Supplemental Videos 1–4).

### BMMSC-IGF1 treatment results in increased remyelination in SCI mice

**Fig. 6:**
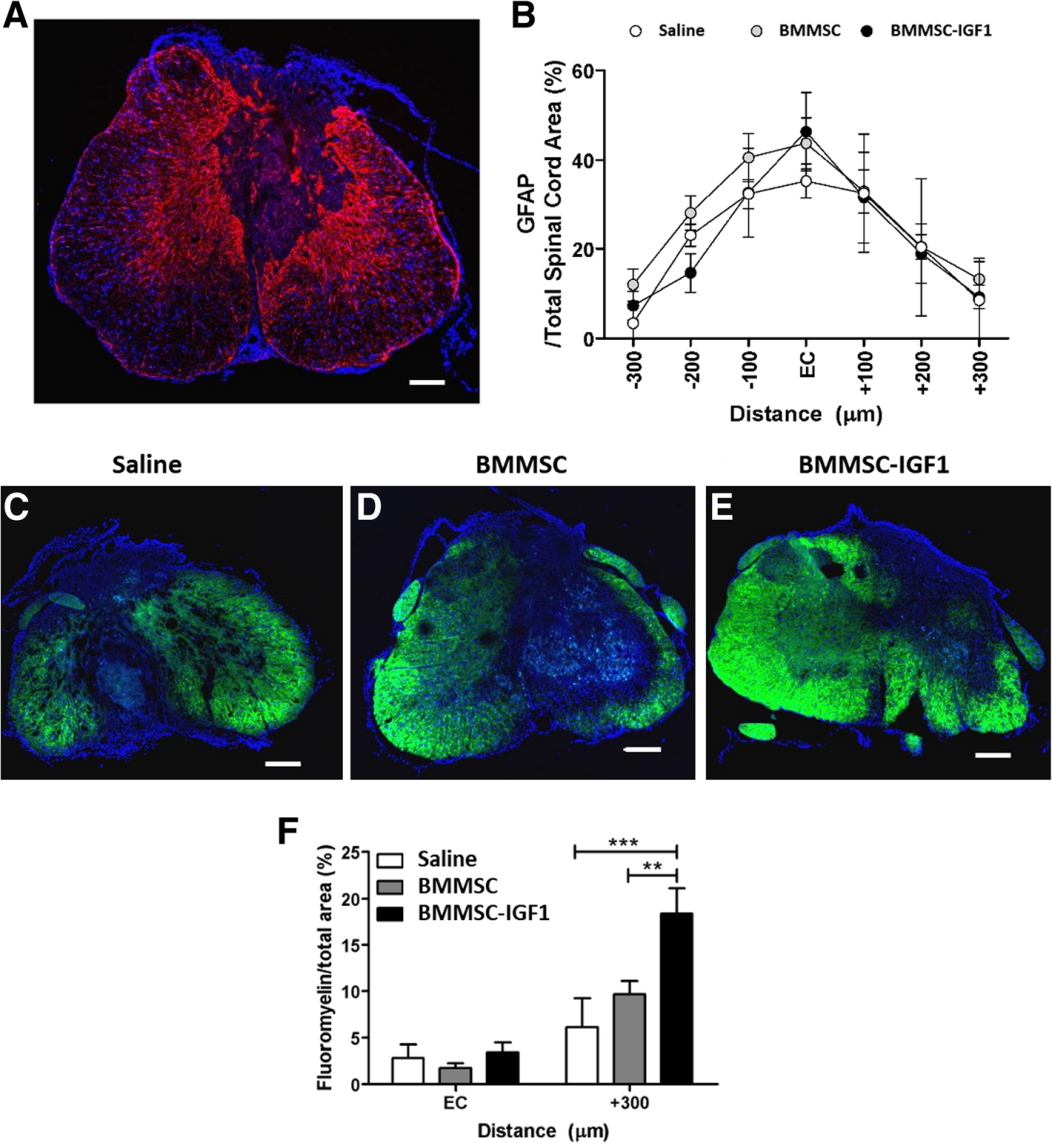
Lesion volume and myelination 4 weeks following SCI and treatment. Lesion volume (**a**) as measured by GFAP staining, which is concentrated around the lesion site, measured against total spinal cord area bilaterally from the epicenter (EC) of the injury. **b** Quantification of GFAP derived lesion volume. Myelination immunostaining with fluoromyelin was performed in saline (**c**), BMMSC (**d**), and BMMSC-IGF1 (**e**) treated SCI sections. Quantitative analysis (**f**) of fluoromyelin-labeled oligodendrocytes at the EC and 300 μm caudally. Bars represent means ± SEM of five mice/group. Values represent mean ± SEM. *P < 0.05 (BMMSC-IGF1: EC vs + 300), ^#^*P* < 0.05 (+ 300: BMMSC-IGF1 vs BMMSC)

Lesion volume of SCI was assessed at the conclusion of the study, 28 days following SCI and treatment. Spinal cord sections were stained for GFAP, in order to evaluate and compare lesion volume between the groups. There was no statistical difference between saline, BMMSC, or BMMSC-IGF1 at the injury epicenter, rostrally or caudally (300 μm from the epicenter, in 100 μm intervals) (Figs. 6a, b).

Myelination was evaluated by fluoromyelin staining (Figs. 6c–e). Similar myelin staining was detected in all groups in the epicenter; however, fluoromyelin-positive staining was significantly higher in SCI-BMMSC-IGF1 mice in slices 300 μm from the epicenter, compared to BMMSC and saline-treated (Fig. 6f).

**Fig. 7:**
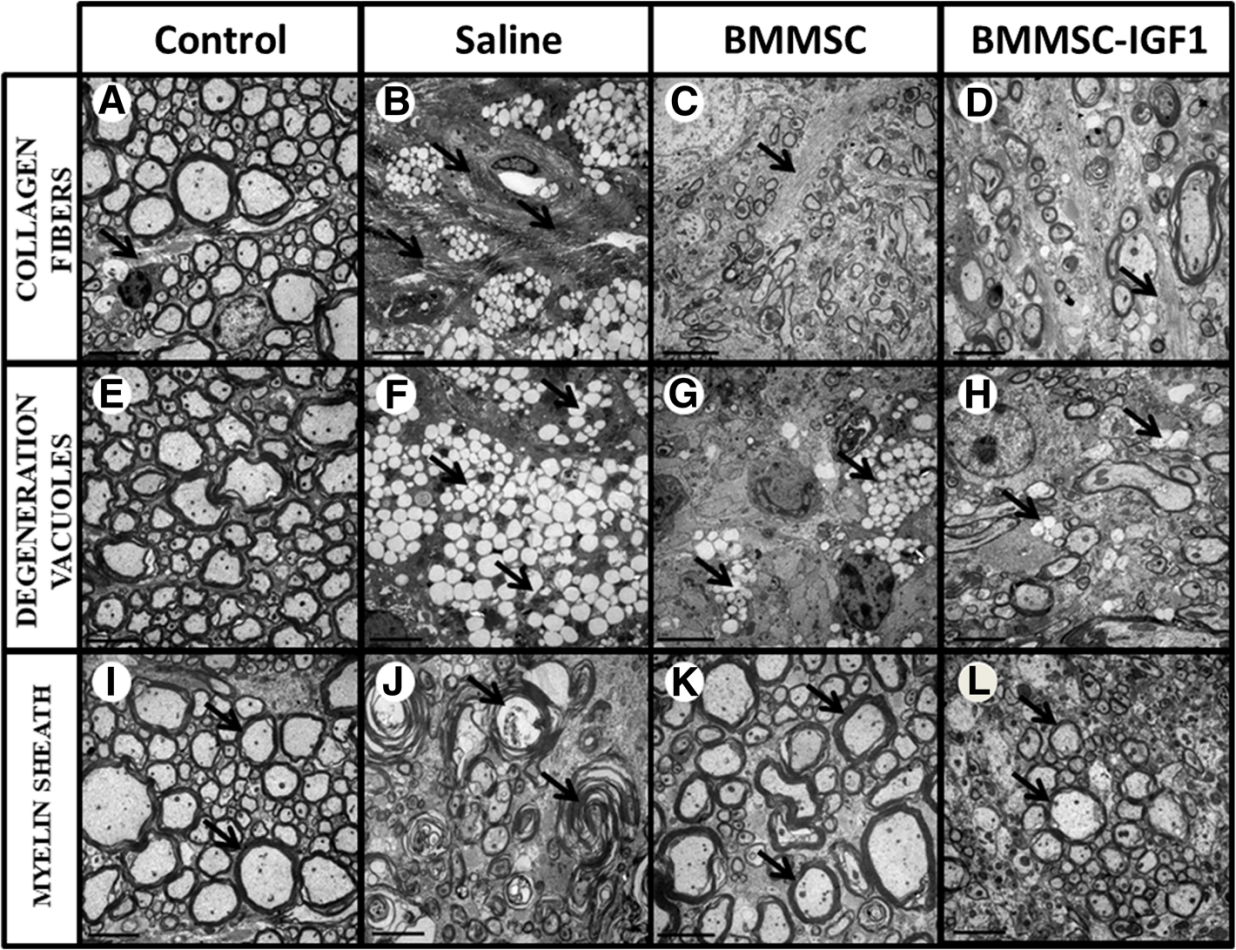
Ultrastructural changes at the site of lesion in animals with SCI visualized by transmission electron microscopy. Representative images of spinal cord sections obtained from uninjured or SCI-mice admistered with saline, BMMCs or BMMC-IGF1 are shown. Morphological patterns of collagen fibers (**a**-**d**), degeneration vacuoles (**e**-**h**) and myelin sheath (**i**-**l**) were evaluated, as indicated by black arrows. Scale bar = 5 μm

In order to confirm the finding of increased myelination in the BMMSC-IGF1 group 28 days post-SCI, we performed transmission electron microscopy to assess ultrastructural morphology in the spinal cord from uninjured, saline-treated, BMMSCs-treated, and BMMSC-IGF1-treated mice. Saline-treated spinal cords presented more dense collagen bundles (Fig. 7b), higher number of degeneration vacuoles (Fig. 7f), and higher number of degenerating axons with myelin sheath collapse (Fig. 7j) when compared to BMMSC(Fig. 7c, g, and k) or BMMSC-IGF1-treated animals (Fig. 7d, h, and l). Conversely, nerve axons-containing preserved myelin sheaths (Fig. 7k, l) and loosely organized, thin collagen fibers (Fig. 7c, d) were more frequently observed in BMMSC- and BMMSC-IGF1-treated animals (Table 1).

**Table 1:** Morphological analysis of the spinal cord of mice 4 weeks after injury

	Saline#1	Saline#2	Saline#3	BMMSC#1	BMMSC#2	BMMSC#3	BMMSC-IGF1#1	BMMSC-IGF1#2	BMMSC-IGF1#3
Collagen fibers	††	†††	†	†	†	†	†	–	†
No. of images	5/10	10/10	2/10	3/10	2/10	1/10	7/10	0/10	1/10
Thickness of the fibers	Thick	Thick	Thin	Thin	Thin	Thin	Thin	–	Thick
Myelin sheath	†	–	††	†††	†††	††	†††	†††	†††
No. of images	10/10	0/10	10/10	8/10	10/10	10/10	9/10	10/10	9/10
Profile	Demyelinated	–	Demyelinated	Myelinated	Myelinated	Myelinated	Myelinated	Myelinated	Myelinated
Degeneration vacuoles	††	†††	†††	†††	–	†	†	–	†††
No. of images	2/10	9/10	1/10	3/10	0/10	6/10	3/10	0/10	1/10

† = little, †† = reasonable, ††† = very

The impact of trauma on axonal myelination revealed a greater demyelinated axon profile in saline-treated mice, while more myelinated axons were observed in BMMSC-and BMMSC-IGF1-treated groups (Fig. 7; Table 1). The pattern of myelin and axons of BMMSC-IGF1 (Fig. 7d) was similar to that observed in uninjured mouse spinal cords (Fig. 7a), with the presence of compact myelin and fewer demyelinated axons.

**Fig. 8:**
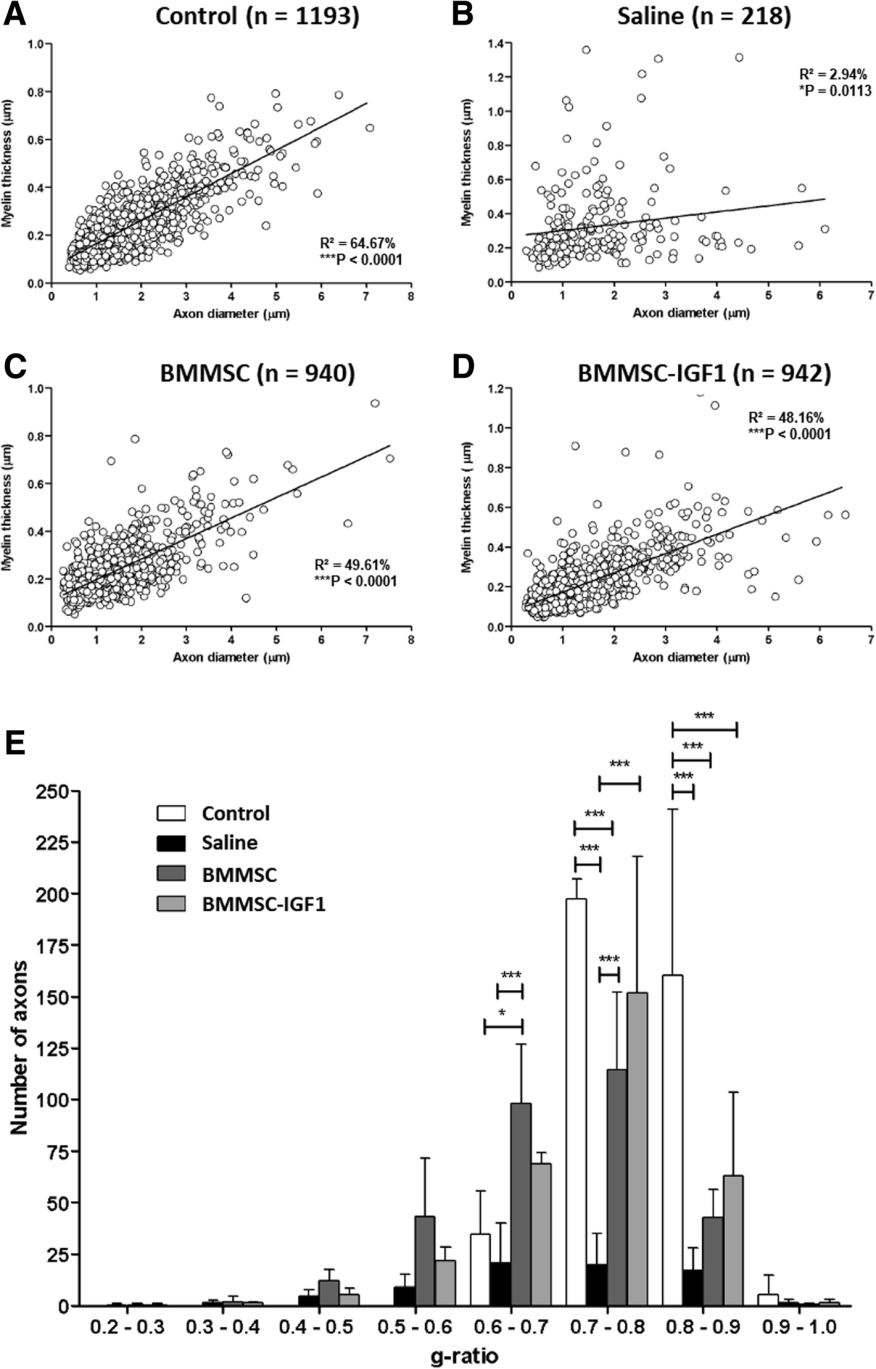
Quantitative ultrastructural analyses of spinal cord sections in uninjured and SCI mice. **a**-**d** Linear regression analysis between myelin thickness and axon diameter from control, saline, BMMSC, and BMMSC-IGF1 groups. Three animals were used in each group. **e** Axon distribution by g-ratio range in control (*N* = 1193), saline (*N* = 218), BMMSC (940), and BMMSC-IGF1 (942) groups. Analysis was done as described in the “Materials and methods” section. Values represent the mean ± SEM in each range. *P < 0.05; ***P < 0.001

We also observed a significant reduction in the total number of myelinated axons from spinal cords with saline treatment (*n* = 218) when compared to uninjured (*n* = 1193), BMMSC-IGF1 (*n* = 942), and BMMSC (*n* = 940). Linear regression of morphometrical analysis parameters demonstrated that BMMSC (Fig. 8c) and BMMSC-IGF1 (Fig. 8d) treated spinal cord lesions presented a similar profile to uninjured mice than saline-treated mice (Fig. 8a). Finally, analysis of g-ratio distribution by range (Fig. 8b) showed a significant difference between both BMMSCs-treated groups when compared to saline animals.

## Discussion

In the present study, we demonstrated that IGF-1 overexpression increases the therapeutic potential of BMMSCs in a moderate contusion SCI mouse model treated during the acute injury phase. We observed that therapy with wild-type BMMSCs led to changes in different parameters evaluated, usually in lower magnitude than what was observed in mice treated with BMMSC-IGF1. However, functional gains were found to be statistically significant only for the group treated with BMMSC-IGF1. This could be the result of direct actions of locally released IGF-1, a growth factor known to participate in the development of the central nervous system as well as in neurogenesis and repair following injury [20, 21]. It is also possible that other intrinsic paracrine actions exerted by BMMSCs were sustained and even enhanced by the increased survival of BMMSC-IGF1 observed in the injured spinal cord.

The acute phase of SCI is characterized by an inflammatory response, which produces a hostile microenvironment during this period [22, 23]. It has been demonstrated that BMMSCs have a very short life span after injection due to several factors, including inflammation and increased oxidative stress [24]. Also, after transplantation, BMMSCs are submitted to an environment of very low glucose levels, near-anoxia, and fail to adapt their metabolism in these conditions, which leads to a rapid depletion of energy reserves and poor survival [5]. In the present study, we demonstrated that IGF-1 overexpression in BMMSCs increases cell survival rate after transplantation to the injured spinal cord, which can be explained by the known pro-survival and anti-apoptotic actions of IGF-1 [25–28]. It is also possible that IGF-1 could act by altering the metabolism of BMMSCs by conferring higher energetic reserves.

Additionally, we observed an increased number of proliferating cells in the spinal cord after treatment with BMMSC-IGF1 and increased number of Iba1^+^ in the BMMSC-IGF1 group. IGF-1 acts as a mitogen for microglia/macrophages in the central nervous system (CNS) [9]. Microglia play an important role in CNS inflammation, as well as in adult neurogenesis. Microglia activation can be detrimental to the survival of newly formed CNS neurons, due in part to the pathological microenvironment [20, 21], but it may be also beneficial, supporting adult neurogenesis and myelination [29]. One such mechanism that microglia has been reported to act benevolently is by mediating an increase in IGF-1 levels, which was shown to lead to neurogenesis and cell proliferation [21, 30].

Increased numbers of DCX^+^ cells were also observed in the group treated with BMMSC-IGF1, suggesting that transplantation of BMMSC-IGF1 recruited endogenous progenitor cells to the injury site. Previously, IGF-1 has been demonstrated to stimulate the production of stromal cell-derived factor (SDF-1) and SDF-1 signaling through CXCR4 leading to protection of neural progenitor cells against hypoxia [31] and is a crucial factor for neural progenitor cells stemness [32]. Additionally, the IGF-1 protein has been reported to promote differentiation by directly binding to IGF-1 receptors on neural stem cells [33]. These data suggest that BMMSC-IGF1 may support the recruitment and maintenance of neural progenitors to the spinal cord following injury, which may participate in the recovery after SCI.

The improved functional outcomes in SCI BMMSC-IGF1 mice appears to be significantly attributed to their effectiveness in minimizing the secondary injury damage to axons and oligodendrocytes, by reduction of oxidative stress. These spared CNS cells were better preserved, as observed by electron microscopy, especially at the g-ratio range of 0.7–0.8, considered to be the optimal value of myelinated fibers [34] and ultimately a more favorable functional recovery. We speculate that this myelination is from oligodendrocytes sparing, as neuroinflammation caused by the secondary injury has been shown to be incompatible with myelin sheath survival [35]. Indeed, increased expression of markers for oligodendrocyte progenitor cells were measured following cell therapy. IGF-1 was previously shown to promote oligodendrocyte differentiation and survival during normal development [12]. Interestingly, we found that myelin integrity was better preserved and complete functional loss was significantly reduced in mice treated with BMMSC-IGF1. This was associated with increased functional recovery in the BMMSC-IGF1-treated group.

Despite the positive gains that we and others have observed with IGF-1, this cytokine has been reported to participate in tumor formation. If the overexpression of IGF-1 in cell therapy was used clinically, the longevity of this expression could be controlled by the introduction of a suicide gene [36], allowing the elimination of these cells in a desired time, after their usefulness has expired. Though we did not observe any tumor formation with BMMSC-IGF1 treatment, in order to avert potentially risky clinical developments, additional studies investigating this and additional growth factors that are not linked with tumor formation should be further explored.

## Conclusion

The association of BMMSCs and IGF-1 is beneficial for the survival of transplanted cells following SCI, as well as for therapeutic effects, as shown by the modulation of the acute phase and secondary injury seen here, which was shown to have a great impact on the long-term functional gains. Finally, we speculate that BMMSC therapy, in combination with growth factors, like IGF-1, may improve the effectiveness of BMMSC mediating tissue regeneration in SCI.

## Additional files

Additional file 1: **Figure S1.** Experimental design of the study. (TIFF 698 kb)

Additional file 2: **Figure S2.** Still frame images of SCI mice in DigiGait system. Representative images of (A) uninjured, (B) saline (C) BMMSC, and (D) BMMSC-IGF1-treated mice during DigiGait evaluation. (TIFF 2504 kb)

## Abbreviations

BMMSC: Bone marrow-derived mesenchymal stem cells
BMS: Basso Mouse Scale
Casp3: Caspase-3
CNS: Central nervous system
DCX: Doublecortin
DMEM: Dulbecco’s modified Eagle’s medium
GFAP: Glial fibrillary acidic protein
GFP: Green fluorescent protein
Gpx3: Glutathione peroxidase 3
IBA1: Ionized calcium binding adaptor molecule 1
IGF-1: Insulin-like growth factor type 1
MBP: Myelin basic protein
MDA: Malondialdehyde
Nfe2L2: Nuclear factor erythroid 2-related factor 2
NOS2: Nitric oxide synthase 2
Olig1: Oligodendrocyte transcription factor 1
Olig2: Oligodendrocyte transcription factor 2
PFA: Paraformaldehyde
RT-qPCR: Quantitative reverse transcription polymerase chain reaction
SCI: Spinal cord injury
SDF-1: Stromal cell-derived factor
