# Supplementary figures and images for: IGF-1 overexpression improves mesenchymal stem cell survival and promotes neurological recovery after spinal cord injury
Source: Stem Cell Res Ther. 2019 May 21;10:146. doi: 10.1186/s13287-019-1223-z (PMC6530133; doi:10.1186/s13287-019-1223-z)

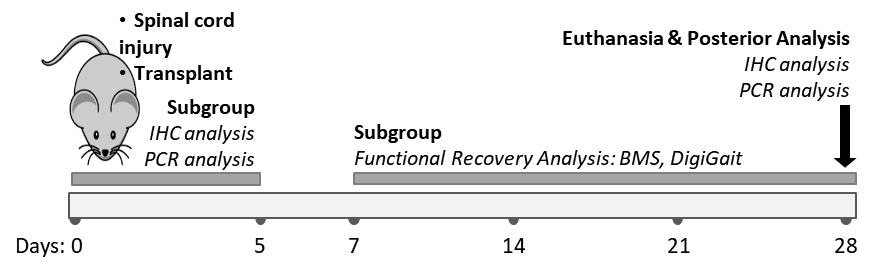

Supplement: Supplementary file 1 — Figure S1. Experimental design of the study. (TIFF 698 kb) [file 13287_2019_1223_MOESM1_ESM.tiff]

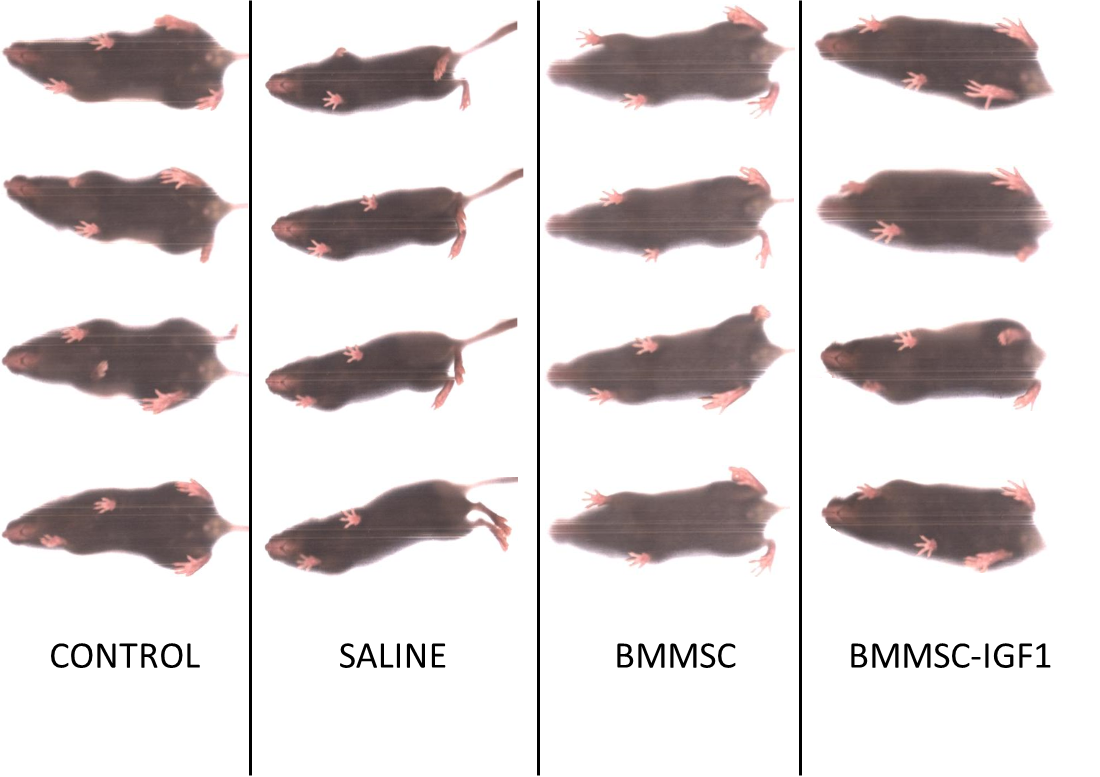

Supplement: Supplementary file 2 — Figure S2. Still frame images of SCI mice in DigiGait system. Representative images of (A) uninjured, (B) saline (C) BMMSC, and (D) BMMSC-IGF1-treated mice during DigiGait evaluation. (TIFF 2504 kb) [file 13287_2019_1223_MOESM2_ESM.tiff]
